# Novel Hyperthermophilic Crenarchaeon *Thermofilum adornatum* sp. nov. Uses GH1, GH3, and Two Novel Glycosidases for Cellulose Hydrolysis

**DOI:** 10.3389/fmicb.2019.02972

**Published:** 2020-01-10

**Authors:** Kseniya S. Zayulina, Tatiana V. Kochetkova, Ulyana E. Piunova, Rustam H. Ziganshin, Olga A. Podosokorskaya, Ilya V. Kublanov

**Affiliations:** ^1^Research Center of Biotechnology, Winogradsky Institute of Microbiology, Russian Academy of Sciences, Moscow, Russia; ^2^Faculty of Bioengineering and Bioinformatics, Lomonosov Moscow State University, Moscow, Russia; ^3^Shemyakin-Ovchinnikov Institute of Bioorganic Chemistry, Russian Academy of Sciences, Moscow, Russia

**Keywords:** cellulase, endoglucanase, glycosidase, cellulose degradation, hyperthermophilic, *Crenarchaeota*, *Thermofilum*

## Abstract

A novel hyperthermophilic, anaerobic filamentous archaeon, *Thermofilum adornatum* strain 1910b^T^, is capable of growing with cellulose as its sole carbon and energy source. This strain was isolated from a terrestrial hot spring in Kamchatka, Russia. The isolate 1910b^T^ grew optimally at a temperature of 80°C and a pH of 5.5–6.0, producing cell-bound inducible cellulases. During genome analysis, genes, encoding various glycosidases (GHs) involved in oligo- and polysaccharide hydrolysis and genes for the fermentation of sugars were identified. No homologs of currently known cellulase families were found among the GHs encoded by the 1910b^T^ genome, suggesting that novel proteins are involved. To figure this out, a proteomic analysis of cells grown on cellulose or pyruvate (as a control) was performed. Both in-depth genomic and proteomic analyses revealed four proteins (Cel25, Cel30, Cel40, and Cel45) that were the most likely to be involved in the cellulose hydrolysis in this archaeon. Two of these proteins (Cel30 and Cel45) were hypothetical according to genome analysis, while the other two (Cel25 and Cel40) have GH3 and GH1 domains, respectively. The respective genes were heterologously expressed in *Escherichia coli* BL21 (DE3), and enzymatic activities of recombinant proteins were measured with carboxymethyl cellulose (CMC), Avicel and cellobiose as substrates. It was revealed that the Cel30 and Cel25 proteins were likely exoglucanases with side beta-glucosidase and endoglucanase activities, that Cel40 was a multifunctional glucanase capable of hydrolyzing beta-1,4-glucosides of various lengths, and that Cel45 was an endoglucanase with side exoglucanase activity. Taking into account that the cellulolytic activity of *T. adornatum* 1910b^T^ surface protein fractions was inducible, that recombinant Cel25 and Cel30 were much less active than Cel40 and Cel45, and that their gene expressions were (almost) non-induced by CMC, we suggest that Cel40 and Cel45 play a major role in the degradation of cellulose, while Cel25 and Cel30 act only as accessory enzymes.

## Introduction

Cellulose is the most abundant organic polymer on Earth; this is why it is a significant part of Earth’s carbon cycle (Maleki et al., 2016). Cellulases (endo-beta-1,4-glucanases) play a crucial role in the degradation of cellulose, and this makes them high-demand enzymes in biomass utilization industries. Endoglucanases are involved in many industrial processes, such as decreasing pulp viscosity in the paper and pulp industry and biopolishing cotton in the textile industry. They are also involved in 2nd generation biofuel production for the enzymatic pretreatment of cellulosic feedstock (Robb et al., 2007; Sims et al., 2010; Kang et al., 2014; Cabrera and Blamey, 2018; Robak and Balcerek, 2018) as well as other economic sectors. Thermostable cellulases offer several benefits in comparison with their mesophilic counterparts. High reaction temperatures decrease the risk of contamination, reduce viscosity, and increase the solubility of substrates, which increases the final yield of the end products (Haki and Rakshit, 2003; Egorova and Antranikian, 2005). Additionally, thermostable enzymes are highly resistant to denaturation and are highly stable during long-term storage, which lengthens both their operational life and their shelf life (Viikari et al., 2007; Peacock et al., 2013).

The following enzymes are the main actors in the hydrolysis of cellulose: endoglucanases/cellulases (3.2.1.4), enzymes that cut internal beta-1,4 glucosidic bonds and resulted in the formation of cellooligosaccharides or cellodextrins, which should be hydrolyzed further by exoglucanases/cellodextrinases (EC 3.2.1.74); beta-glucosidases (EC 3.2.1.21); cellobiose phosphorylases (EC 2.4.1.20); cellodextrin phosphorylases (EC 2.4.1.49); and a few other enzymes (Davies and Henrissat, 1995; Lynd et al., 2002). Among them, endoglucanases/cellulases are of particular significance because their action leads to a considerable reduction in the degree of cellulose polymerization, exponentially increasing the concentration of substrates for other enzymes (Béguin and Aubert, 1994). According to the Carbohydrate-Active Enzymes Database (CAZy^1^), among 165 GH families (as of October 2019), endoglucanases are members of the GH5–9, GH12, GH44, GH45, GH48, GH51, GH74, and GH124 families (Lombard et al., 2014).

*Archaea* are still in the shadow of *Bacteria* in terms of public opinion, the depth of scientific knowledge, and their impact on industrial biotechnology (Straub et al., 2018). There are several reasons for this, among which are the historical development of science, difficulties in isolation and cultivation because the majority of cultivated archaea are still extremophiles and the lack of facile archaeal genetic tools (Straub et al., 2018). Thus, despite the fact that archaea are similar to bacteria in diversity and abundance (Offre et al., 2013), their metabolic variability and industrial potential are lower than those of bacteria. In particular, despite the fact that many bacterial and eukaryal cellulolytic microorganisms have been discovered to date, our knowledge about the degradation of cellulose by archaea is limited. Utilization of cellulose as a growth substrate was demonstrated for several halophilic and natronophilic representatives of the *Natrialbales* and *Halobacteriales* orders (Sorokin et al., 2015, 2018, 2019) and for hyperthermophilic *Thermococcales* (Mardanov et al., 2009; Gavrilov et al., 2016). A few of the *Thermococcus* and *Pyrococcus* cellulases belong to GH5 and GH12 (Kim and Ishikawa, 2010; Nakahira et al., 2013; Kataoka and Ishikawa, 2014; Gavrilov et al., 2016). As for the *Crenarchaeota*, our current knowledge is restricted to two publications that report a relatively weak growth on cellulose for representatives of *Desulfurococcales* (Perevalova et al., 2005; Kochetkova et al., 2016). Surprisingly, in the genomes of these archaea, no genes of known cellulase families were found that hide their cellulose hydrolysis mechanisms (Mardanov et al., 2012; Susanti et al., 2012). On the other hand, cellulase genes were found in the genomes of some other crenarchaeota, for which the growth on cellulose was not shown. For example, several GH12 family endoglucanases from *Sulfolobus solfataricus* and *S. shibatae* were heterologously expressed in *Escherichia coli* (Limauro et al., 2001; Girfoglio et al., 2012; Boyce and Walsh, 2018). Also using sequence-based and functional metagenomics approaches, a few of the endoglucanases from the GH12 family as well as a novel GH representative were obtained from hyperthermophilic microbial consortia (Graham et al., 2011; Leis et al., 2015; Suleiman et al., 2019).

Representatives of *Thermofilaceae* are moderately acidophilic and hyperthermophilic crenarchaea are obligately dependent on various components of cells or culture broths of other *Crenarchaeota*, yet they are capable of utilizing polysaccharides as sources of carbon and energy (Zillig et al., 1983; Toshchakov et al., 2015; Kochetkova et al., 2019). Until now, cellulose was not among the substrates utilized by representatives of the family except for the weak growth on Avicel of *Thermofilum* sp. strain 1505 (Kochetkova et al., 2019). In this work, we demonstrate the novel species *Thermofilum adornatum* sp. nov. strain 1910b^T^’s ability to grow on cellulose, including insoluble Avicel and in production cell-bound inducible cellulases. Genomic and proteomic-based surveys revealed four novel cellulases of various families, which were heterologously expressed in *E. coli*, and their cellulolytic activities were confirmed and compared.

## Materials and Methods

### Culture Characterization and Cultivation

*Thermofilum* strain 1910b^T^ was isolated from a Kamchatkan hot spring in 2009 (Dominova et al., 2013). A strictly anaerobic modified freshwater Pfennig basal medium (Podosokorskaya et al., 2011) was used for routine cultivation and all growth experiments. The addition of at least 0.05 g/l of yeast extract and 1/100 (v/v) of culture broth filtrate (CBF) for other crenarchaea such as *Fervidicoccus fontis* (strain 1910a = UNIQEM 1910a), *Pyrobaculum aerophilum* (strain Kam13-1) or *Desulfurococcus amylolyticus* (strain 1221*n* = DSMZ 18924) was mandatory. The utilization of various organic substrates was tested under standard growth conditions under N_2_ in the gas phase (80°C, pH 5.5). Other *Thermofilum* species, such as *T. uzonense* strain 1807-2^T^ (Toshchakov et al., 2015) and strain 1505 (Kochetkova et al., 2019), were used for comparative growth experiments and were taken from the laboratory collection.

Strain 1910b^T^ was deposited in DSMZ (German Collection of Microorganisms and Cell Cultures) with the designation DSM 28063, and it was also deposited in JCM (Japanese Collection of Microorganisms) with the designation JCM 19809.

### Cloning and Heterologous Expression of the Putative Glycosidase Genes in *Escherichia coli*

Cloning of strain 1910b^T^ genes *Cel25* (2101 bp), *Cel30* (1120 bp), *Cel40* (1558 bp), and *Cel45* (235 bp) was performed using the aLICator Ligation Independent Cloning (LIC) and Expression System kit (#K1251, Thermo Fisher Scientific). The genes were amplified according to Innis and Gelfand (1990) using the *de novo* designed primers (Supplementary Table S1) and strain 1910b^T^’s genomic DNA as the matrix. For the *Cel40* and *Cel25* genes, a nested PCR (Lualdi and Fasano, 2019) was performed because no PCR product with genomic DNA was obtained. This was possibly due to inconsistent theoretical parameters for the use of the respective primer pairs (low GC% content, T melting, etc.). All primers (except for those that were used for nested PCR) include vector-specific sequences (underlined in the table) complementary to linear vector pLATE 51, which contained N-terminal 6x, His-tag and an enterokinase cleavage site (DDDDK^).

The PCR products were purified with the Cleanup Standard Kit (#BC022, Evrogen). The LIC reaction was performed for 5 min at 25°C, upon which the vector with the insert was directly transformed into *E. coli* BL21 (DE3) competent cells. This was done following the instructions of the manufacturer, and a 720 bp control PCR fragment was used to assess the efficiency of the LIC reaction. The presence of cloned genes was confirmed by PCR with DNA from grown *E. coli* colonies as the matrix and specific primers (LIC Forward Sequencing primer 5’TAATACGACTCACTATAGGG and LIC Reverse Sequencing primer 5′GAGCGGATAACAATTTCACACAGG), and this was followed by sequencing (Evrogen).

Recombinant *E. coli* strains containing plasmids with target genes were grown in a Luria-Bertani medium (LB) supplemented with ampicillin (100 μg/ml) and 30 μg/l of chloramphenicol for control. This was induced by adding 1.0 mM of isopropyl-β-D-thiogalactopyranoside (IPTG) to express recombinant proteins to a final concentration of cells corresponding to ∼0.5 at OD 600, and it was then incubated at 25°C for 16 h. A volume of 15 ml of the recombinant cells was harvested by centrifugation at 4,000 *g* for 15 min at 4°C, washed with a 25 mM phosphate buffer (pH 7.5) and resuspended in 0.7 ml of the same buffer with 0.5 M NaCl and 25 mM imidazole. After sonication, the cell extracts were centrifuged (15,000 *g* at 4°C for 15 min), and resulting supernatant, as well as the *E. coli* culture broth, were tested for enzymatic activity. The concentration of the purified protein was determined by the Qubit^TM^ protein Assay Kit (#Q33212, Invitrogen).

### Glycosidase Activity Measurements

#### Native Cellulases

Strain 1910b^T^ was cultivated at optimal growth conditions with 0.5 g/l of microcrystalline cellulose (Avicel) until it reached a cell density of 5–7^∗^10^6^ cells/ml (120 h of incubation). Native glycosidase activities were determined in the culture broth and solution of proteins, which were extracted from cell surfaces (SPF, surface protein fraction) according to Gavrilov et al. (2016). The resulting SPFs were 10-times diluted in 50 mM MES (pH^25°C^ = 5.6) before measurements. The protein concentration was measured using the Qubit^TM^ Protein Assay Kit. Enzyme preparations were mixed with 0.1% (w/v) amorphous cellulose (AMC), prepared according to Sorokin et al. (2015), and 0.1% (w/v) carboxymethyl cellulose (CMC) in 0.05 M MES (pH^25°C^ = 5.6) and incubated at 80°C for 90 h. Aliquots of the reaction mixture were sampled every 20 h and stored at 4°C before activity measurements were taken. Glycosidase activities were determined using a DNS assay (Miller, 1959). D-glucose solutions of various (50–500 μg/ml) concentrations were used to plot a calibration curve. One unit (U) of enzyme activity was defined as the amount of enzyme required to release 1 μmol reducing sugar (glucose) in minute at 80°C under the described conditions. Specific activity was defined as enzyme activity per milligram of protein (U/mg).

#### Recombinant Cellulases

A qualitative analysis of endoglucanase activities was performed as follows: 50 μl of crude extracts of *E. coli* BL21 (DE3) with recombinant proteins were put into the wells in the agar plates, which contained 2% (w/v) agarose and 0.2% (w/v) CMC as a substrate. The plates were incubated at 80°C for 16 h, and this was followed by staining with 0.2% (w/v) Congo red for 30 min (5 ml for each plate) and destaining with 1 M NaCl three times for 15 min at room temperature. Crude extracts of *E. coli* BL21 (DE3) with empty vectors were used as the control experiment.

Quantitative measurements of cellulolytic and cellobiase activities were performed using the DNS method (see above) and CMC and cellobiose as substrates. The reaction mixtures contained 1980 μl of 0.2% (w/v) substrates in a MES buffer (50 mM, pH 5.6) and 20 μl of recombinant enzymes from crude extracts. The reaction mixtures incubated at the optimal temperature (80°C) for 4 h. Crude extracts of *E. coli* BL21 (DE3) with empty vectors were used as the control experiment.

Thin-layer chromatography (TLC) was used to determine the products of cellulose and its derivative hydrolysis. An incubation of recombinant enzymes, which were prepared with CMC, AMC or cellobiose [1% (w/v) each] as the substrates, was performed the same way as it was for the DNS assay (see above). The products of hydrolysis were separated on an aluminum sheet (20 cm × 20 cm) and 60 silica gel plates (Merck), upon which a solution of butanol, ethanol and H_2_O (2:2:1, v/v/v) was added. The separation of hydrolysis products was performed in a Latch-lid^TM^ TLC developing chamber. A solution containing glucose, cellobiose, cellotriose, cellotetraose, cellopentaose, and cellohexaose [0.0625% (w/v) each] was used as the marker. After elution, the plates were dried at 42°C for 30 min and then dried again at 65°C for 20 min. Mono- and oligomers were observed upon spraying the plates with a 0.1% orcinol solution in 5% (v/v) H_2_SO_4_. This was followed by drying at 75°C for 10–15 min.

### Functional Genome Analysis

The genome of strain 1910b^T^ was sequenced in another work (Dominova et al., 2013). The genome was deposited in Genbank under the accession numbers CP006646 and IMG 2554235458. The reconstruction of metabolic pathways was done using the KEGG (Kanehisa et al., 2016) and MetaCyc (Caspi et al., 2014) databases. The predicted protein functions were analyzed using a hidden Markov model-based (HMM-based) homology search via the HMMER web server^2^ against the Pfam 27.0 database (Finn et al., 2014) and dbCAN 2.0 web resource (Yin et al., 2012; Lombard et al., 2014) was analyzed against the CAZy database. Transporter predictions were done using a BLAST-search against the transporter classification database TCDB (Saier et al., 2014). A more sensitive search for better function prediction was performed using PSI-BLAST (Altschul et al., 1997), genome context analysis and the presence of specific regulatory sequences. Signal peptides were predicted with the SignalP 4.1 (Petersen et al., 2011) and TatP 1.0 servers (Bendtsen et al., 2005); transmembrane helices were predicted with the HMM-based TMHMM 2.0 web server (Krogh et al., 2001). The HMM-based server that combined transmembrane protein topologies and signal peptide predictors, Phobius (Käll et al., 2004), was also used to verify the respective predictions.

### Proteomic Analysis

For proteomic analysis, strain 1910b^T^ was grown under optimal growth conditions (80°C and a pH of 5.7) with 1 g/l of Avicel until it reached a cell density of 5–7^∗^10^6^ cells/ml. The experiment was done in four biological replicates (4 bottles that each contained 1.2 l of culture). The control growth experiment (1 g/l of pyruvate instead of cellulose as a substrate), was made in three replicates. The grown cells were collected by centrifugation at 17,600 *g* for 20 min and lyzed according to Kulak et al. (2014). The protein concentration in the cell-free culture broth was under the detection limit when measured using a Brandford reagent. Cell proteins (both intracellular and cell-wall) were treated the same way as described by Kulak et al. (2014). NanoLC-MS/MS analysis was performed as described previously (Sidorenko et al., 2018). Raw MS data were analyzed using the MaxQuant software package^3^ against the strain 1910b^T^ genomic sequence (CP006646). Following this, proteomic analysis was conducted using R. The script is available in the repository^4^.

Intensity-based absolute quantitation (iBAQ) quantitative values of detected proteins were obtained and normalized as summed iBAQ values within one sample, which represent the molar abundance or relative iBAQ (riBAQ) of an identified protein within a sample. For each detected protein, the log2 riBAQ value was calculated (Cijsouw et al., 2018). To determine the similarity of the samples’ expression profiles, a hierarchical clustering of the samples based on the correlation coefficients of the riBAQ values was performed. To determine the genes with statistically significant differences in expression, an independent, two-sample *t*-test with the Benjamini–Hochberg correction was used (Green and Sambrook, 2019).

## Results

### Cell Morphology and Growth Characteristics

**FIGURE 1:**
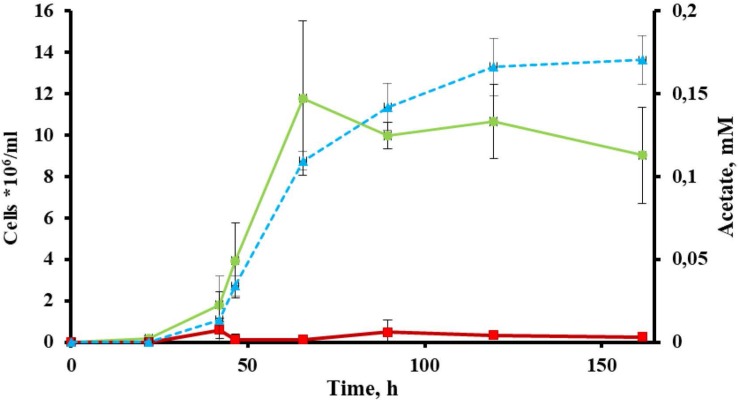
Growth of *Thermofilum adornatum* strain 1910b^T^ on cellulose. Cell yield of 1910b^T^ grown on basal medium containing 0.05 g/l yeast extract (red) or 0.5 g/l amorphous cellulose (green). Concentration of acetate (blue), producing during strain 1910b^T^ growth on cellulose. The incubation temperature was 80°C and the pH of the media was 5.6.

Cells of strain 1910b^T^ were thin, straight filaments 0.15 μm in width and 2–20 μm in length. One sub-polar flagellum was occasionally observed (data not shown). Strain 1910b^T^ was an obligate anaerobe, growing optimally on glucose at a temperature of 80°C and at a pH of 5.5–6.0. The generation time and maximum cell density under optimal growth conditions with glucose as a substrate were ∼5 h and ∼5^∗^10^7^ cells/ml, respectively. The isolate grew on carbohydrates such as starch, β-glucan, Avicel and AMC (Table 1) with a final cell yield of 5–15^∗^10^6^ cells/ml (Figure 1).

**TABLE 1:** Phenotypic characteristics of *Thermofilum* strains.

**Characteristics**	***T. adornatum* 1910b^T^**	***Thermofilum* sp. 1505**	***T. pendens* Hvv3^T^**	***T. uzonense* 1807-2^T^**
Isolation source	Kamchatka	Kamchatka	Iceland	Kamchatka
Min./opt./max. growth temperature (°C)	50/80/95	85	85–90	70/85/90
Min./opt./max. pH for growth	5.3/5.5–6.0/8.5	6.8	4.0/5.0–6.0/6.5	5.5/6.0–6.5/7.0
Growth factors dependence	*Desulfurococcus*, *Pyrobaculum* or *Fervidococcus* CBF, and yeast extract	*Desulfurococcus* CBF, yeast extract, and cysteine	*Thermoproteus tenax* polar lipid fraction and yeast extract	*Desulfurococcus* or *Pyrobaculum* CBF and yeast extract
S^0^-dependence	−	−	+	−
Utilized substrates				
AMC	+	−	(+)	−
Avicel	+	+		−^∗^
α-cellulose	−	−		ND
CMC	−	−		−
Filter paper	−	−		−
β-glucan	+	ND	ND	ND
Starch	+	ND	(+g)	+
Mannan	−	ND	ND	−
Glucomannan	−	ND	ND	+
Cellobiose	+	−	ND	−^∗^
Glucose	+	ND	(+g)	+
Sucrose	−	ND	+	ND
Maltose	−	ND	ND	−^∗^
Lactose	+	ND	ND	ND
Mannose	+	ND	ND	−^∗^
Pyruvate	+	ND	ND	ND
G + C mol%	46.5	46.4	57.6	47.9
Genome (Mb)	1.75	1.75	1.78	1.61
Reference	This work	Kochetkova et al., 2019	Zillig et al., 1983; Anderson et al., 2008	Toshchakov et al., 2015

*All strains are strict anaerobes and capable of fermenting yeast extract and peptone. ND, no data available; +, growth; −, no growth (no difference with the control); (+g), the genes, encoding enzymes, presumably involved in degradation of these substrates were found by genome analysis; ^∗^, our data.*

The phylogenetic position of strain 1910b^T^ as well as the average nucleotide identity (ANI) values between 1910b^T^ and validly published *T. pendens* and *T. uzonense* placed strain 1910b^T^ in a separate species of the genus (Kochetkova et al., 2019). Taking into account the phenotypic properties of 1910b^T^ (cellulose, β-glucan, cellobiose, mannose, lactose and pyruvate utilization), we propose a novel species of the genus, *T. adornatum* sp. nov., with the type strain 1910b^T^. A description of the new species is given below.

### Native Glycosidase Activity

To reveal its native cellulase activity, *T. adornatum* 1910b^T^ was grown on Avicel for 5 days under optimal growth conditions. Upon centrifugation (17,600 *g* for 20 min), the cell-free culture broth and cell pellet fractions were incubated with CMC or AMC, and the formation of reduced sugars was measured using the DNS approach. Cellulolytic activity was detected only in the cells with a reducing sugars formation rate of 19.33–49.45 μmol glucose/ml/minute (U) (Table 2). The cell wall linked cellulases were washed from the cells’ surfaces by treatment with solutions of urea, Triton-X100, Tween-80 and SDS. All except the SDS-washed solutions of surface protein fractions (SPFs) contained active cellulases (Table 2).

**TABLE 2:** Native cellulases activity of *T. adornatum* 1910b^T^.

**Fraction**	**Enzyme activity, U**	
	**AMC**	**CMC**
Cells	19.33	49.45
Supernatant	0	0
SPF (SDS)	0	0
SPF (Triton-X100)	18.7	20.4
SPF (Urea)	21.39	37
SPF (Tween-80)	8.16	9

**FIGURE 2:**
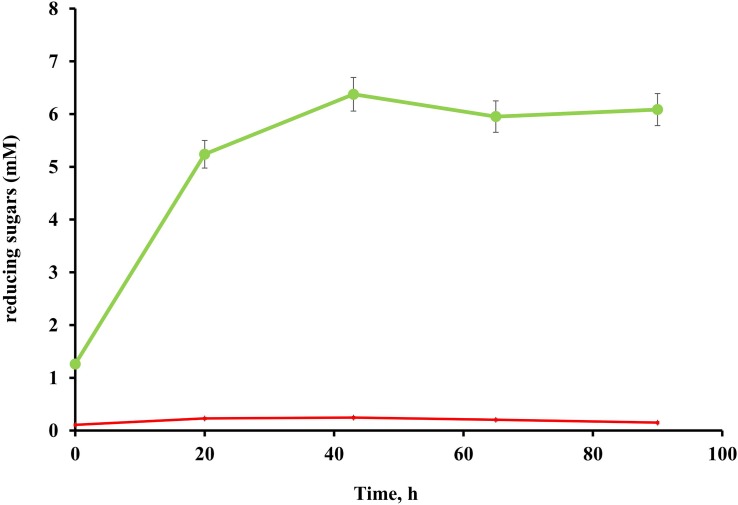
Endoglucanase activity of *T. adornatum* 1910b^T^, grown on Avicel (green), or pyruvate (red). The protein concentrations were normalized between the experiments. In both cases the strain was grown under optimal growth conditions. For enzymatic activity measurements CMC was used as the substrate, the incubation time was 90 h, temperature and pH were 80°C and pH 5.6, respectively.

No cellulolytic activity was detected in either the culture broth or cell pellet fractions of *T. adornatum* 1910b^T^ grown on pyruvate, suggesting that cellulases are cellulose inducible (Figure 2).

### Genomic Analysis of Sugar Metabolism

The genome of *T. adornatum* 1910b^T^ contained 39 genes, which were predicted to encode CAZymes (Lombard et al., 2014). Seventeen of them encoded putative glycosidases (GH), 16 encoded glycosyl transferases (GT), 5 encoded carbohydrate esterases (CE), and 1 encoded a protein with auxiliary activities (AA). Four putative GHs, encoded by *N186_RS00270* (GH113), *N186_RS08040* (GH57), *N186_RS08045* (GH13), and *N186_RS08615* (GH16), were predicted to be extracellular proteins. The remaining putative GHs were predicted to be intracellular because no signal peptides were predicted by SignalP, TatP or Phobius (Supplementary Table S2). N186_RS08040 with GH57 and N186_RS08045 with GH13 domains were probably involved in starch degradation. Their genes were located in the cluster *N186_RS08010-8060*, also encoded various subunits of ABC transporters, possibly involved in the import of maltooligosaccharides. Another putative GHs of the GH57 family (*N186_RS01555* and *N186_RS01850*) probably participated in the intracellular hydrolysis of maltooligosaccharide or glycogen. Three proteins that presumably participated in both alpha-mannan degradation and utilization were identified. The genes of two of them (*N186_RS07230* and *N186_RS07265*) were co-located with the sugar-specific transcriptional regulator *TrmB* (*N186_RS07255*), while the gene of putative alpha-mannosidase (*N186_RS07400*), which contained two GH38 domains, was clustered with the gene of hypothetical fructose-bisphosphate aldolase (*N186_RS07295*). The close location of the carbohydrate ABC transporter and sugar permease (*N186_RS07240*, *N186_RS07245* and *N186_RS07250*) genes suggest the co-action of all these proteins in alpha-mannan degradation. The following proteins were predicted to participate in beta-linked sugar hydrolysis including cellulose and its derivatives: a GH1 glycosidase (*N186_RS00340*, *Cel40*) which gene was clustered with ABC transporters and permease genes *N186_RS00315*, *N186_RS00320*, *N186_RS00325*, and *N186_RS00330*. Another GH1 (*N186_RS06555*, putative beta-galactosidase) gene was co-located with the genes, encoding a PTS transporter (*N186_RS06510*), a sugar-phosphate permease (*N186_RS06540*), and a galactokinase (*N186_RS06575*). The genes of three hypothetical proteins, one of which was homologous to transporters (*N186_RS00220*), another – sugar-specific transcriptional regulators (*N186_RS00235*), while the third one *N186_RS00230* (*Cel30*) lacks any detectable characterized homologs were co-located with a gene, encoded a putative β-glucosidase (*N186_RS00225*, *Cel25*) of the GH3 family. A gene of putative endo-1-3-β-glucanase (*N186_RS08615*) containing a GH16 domain was located near two genes, encoding hypothetical protein N186_RS08625 and DUF86-containing N186_RS08645. No genes that encoded GHs of families contained biochemically characterized cellulases were found in the 1910b^T^ genome.

The central carbohydrate metabolism of *T. adornatum* 1910b^T^ was similar to other hyperthermophilic archaea (Bräsen et al., 2014). Glucose utilization occurred via the archaeal type Embden–Meyerhof–Parnas (Ahmed et al., 2005), for which all necessary genes were found. The first reaction of this pathway involves hexokinases of the ROK family N186_RS05865 which phosphorylates hexoses such glucose and mannose. Isomerization of glucose-6-phosphate into fructose-6-phosphate was catalyzed by phosphoglucose/phosphomannose-isomerase N186_RS04475, which is capable of using both glucose and mannose as substrates. Phosphofructokinase N186_RS07290 of *T. adornatum* 1910b^T^ is probably an ATP-dependent enzyme belonging to the PFK-B family, which representatives are known to be distributed among *Crenarchaeota* (Bräsen et al., 2014). *Thermofilum*’s fructose-1,6-bisphosphate aldolase N186_RS07295 belongs to the family of the archaeal type class I aldolases. The oxidation of glyceraldehyde-3-phosphate occurred in a one-step, irreversible reaction catalyzed by GAPOR (ferredoxin-dependent glyceraldehyde-3-phosphate oxidoreductase N186_RS1755, N186_RS8210, N186_RS1345, and N186_RS0640).

### Proteomic Analysis

Despite the fact that *T. adornatum* 1910b^T^ grew on cellulose as a sole energy and carbon source for synthesizing cell-bound cellulases, no currently known cellulase-encoding genes were found in its genome. To reveal the proteins involved in cellulose degradation and cellulases in particular, strain 1910b^T^ was grown on Avicel or pyruvate (as the control), followed by LC-MS/MS proteomics. Analysis of experimental and control proteomes revealed 139 of 1215 genes, the expression of which was higher on cellulose than on pyruvate (109 genes were downregulated). Most of the upregulated genes (84 of 139) encoded hypothetical proteins, ABC transporters, permeases, and other transporters and proteins involved in ribosome and nucleotide biosynthesis. Thirteen, eight and four proteins encoded by upregulated genes were predicted to be secreted by Phobius, TatP, and SignalP, respectively (Supplementary Table S3). The majority (12 of 17) of glycosidase genes were non-regulated or even downregulated on cellulose (Supplementary Table S4). Among upregulated genes, three (*N186_RS08540*, *N186_RS08045* and *N186_RS01850*) were predicted to encode enzymes, capable of hydrolyzing maltose, starch and its derivatives; two (*N186_RS07750* and *N186_RS04390*) were glycosyl transferases with a predicted inverting mechanism (thus, they were irreversible); and only one protein (N186_RS00340, Cel40) was annotated as a representative of the GH1 family, which means that it was possibly involved in beta-glucans hydrolysis.

**FIGURE 3:**
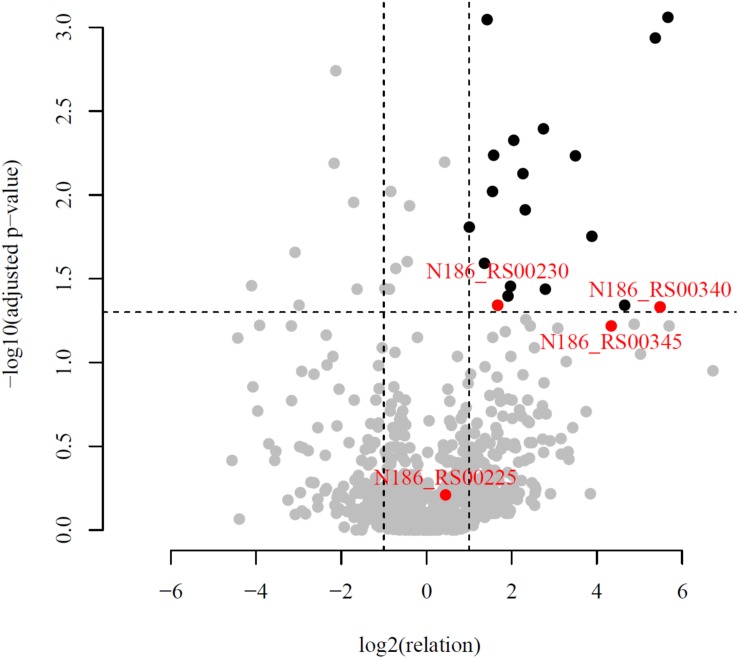
Volcano plot of all quantified proteins. Horizontal line corresponds to the adjusted *p*-value = 0.05. The plot showed the -log10 of the adjusted *p*-value against the log2 of the fold change. Proteins with statistically (*p*-value < 0.05) and biologically (fold-change > 2) significant differences in expression are colored in black and situated on the right top. The proteins with abscissa values more than 2^6^ and ordinate values more than 10^–3^ are not shown but present in the Supplementary Table S3. Red dots correspond to candidate proteins, participating in cellulose degradation.

An in-depth genomic analysis of these upregulated genes and their genomic context revealed four candidates [*Cel25*, *Cel30*, *Cel40*, and *N186_RS00345* (*Cel45*)] (Table 3), which were the most likely to be involved in cellulose hydrolysis. The selection of putative cellulases was based on a combination of the following criteria: (1) a high ratio of experimental (cellulose) riBAQ values to the control (pyruvate) riBAQ values (riBAQ_cel./riBAQ_contr., Figure 3), (2) the presence of detectable GH domains of families with known activity against β-linked carbohydrates, (3) extracellular localization (predictions based on signal peptide and N-terminal transmembrane helix) and (4) the gene neighborhood with genes that encoded proteins involved in carbohydrate metabolism. It should be noted that the chosen genes should not necessarily need to fit all of the criteria.

**TABLE 3:** Candidate proteins, proposed to be involved in cellulose hydrolysis by *T. adornatum* 1910b^T^.

**Gene ID**	**riBAQ_cel/riBAQ_contr**	**Function**	**Pfam**	**Localization**	**Gene length, bp**
*N186_RS00340* (*Cel40*)	44.8	Glycoside hydrolase family 1 protein	GH1	Intracellular	1558
*N186_RS00345* (*Cel45*)	11.3	Hypothetical protein	No hits	Intracellular	235
*N186_RS00230* (*Cel30*)	3.2	Hypothetical protein	No hits	Extracellular	1120
*N186_RS00225* (*Cel25*)	1.0	Glycoside hydrolase	GH3	Intracellular	2101

### Glycosidase Activity of the Recombinant Proteins of *Thermofilum adornatum* 1910b^T^

The four selected genes were heterologously expressed in *E. coli* BL21 (DE3) using the pLATE expression system (pLATE51 with N-terminal His_6__x_-tag). After the destruction of *E. coli* cells and centrifugation of cell debris, only Cel45 was active on the agar plate with CMC as a substrate (Supplementary Figure S1).

**FIGURE 4:**
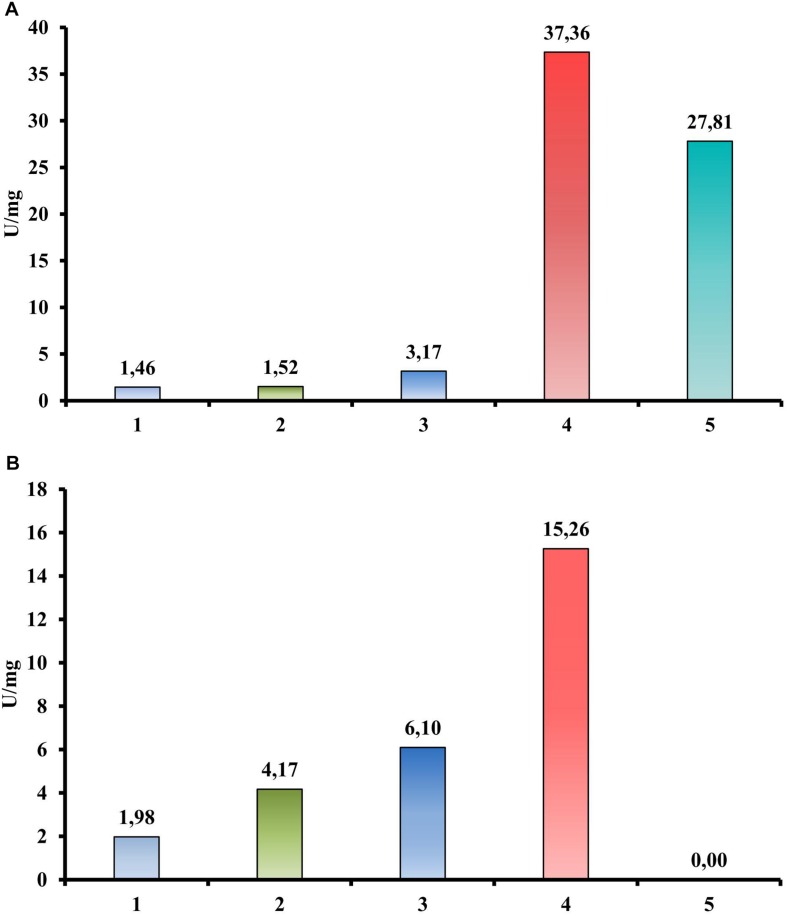
Hydrolytic activities of recombinant proteins from *T. adornatum* 1910b^T^, measured by DNS. **(A)** CMC is a substrate. **(B)** Cellobiose is a substrate. 1, crude extract of *Escherichia coli* cells with empty vector (control); 2, crude extract of *E. coli*, containing Cel25; 3, crude extract of *E. coli*, containing Cel30; 4, crude extract of *E. coli*, containing Cel40; 5, crude extract of *E. coli*, containing Cel45. Incubation: 3 h at 80°C.

In contrast, a quantitative activity measurement using a DNS assay with CMC as a substrate revealed the endoglucanase activity of the Cel40, Cel45, and Cel30 proteins (Figure 4A). Moreover, the most active enzyme was Cel40 (37.36 U/mg). The same experimental setup using cellobiose instead of CMC as a substrate (Figure 4B) showed three proteins (Cel40, Cel30 and Cel25) that possessed beta-glucosidase activity (15.26, 6.10, and 4.17 U/mg, respectively).

### Thin-Layer Chromatography Analysis of Cellulose and Cellobiose Hydrolysis Products

The TLC of the products of CMC hydrolysis by the four studied proteins revealed the production of glucose (C1), cellobiose (C2), and cellotriose (C3) by Cel40 and the production of C2–C3 by Cel25 and Cel30. In its turn, Cel45 produced no detectable C1–C6 products (Supplementary Figure S2). Analysis of the AMC hydrolysis products revealed a formation of C1 and C2 due to the action of the Cel40, no detectable C1-C6 sugars were observed after incubation of AMC with three other enzymes. Hydrolysis of cellobiose was observed only for Cel40 and Cel25 (Supplementary Figure S2).

## Discussion

The most extremophilic and thermophilic microorganisms are represented by archaea. Despite high demand, linked with their extreme resistance to harsh conditions, archaea still cannot be regarded as key players in bioindustry for several reasons (see section “Introduction”). Yet some progress has been made recently, and this progress is mainly linked with the development of novel archaeal genetic tools (Straub et al., 2018) and the progress in isolation of novel archaea. The latter is one of the crucial (Straub et al., 2018) yet difficult steps toward the involvement of archaea in bioindustry. An isolation of novel extremophilic archaea, especially those of deep phylogenetic lineages and/or possessing rare or novel properties, gives us a good opportunity to develop novel technologies or improve current ones. The crenarchaeal family *Thermofilaceae* is a deeply branching lineage within the order *Thermoproteales*, which consists so far of only one genus (*Thermofilum*). This genus has only two validly published species, *T. pendens* and *T. uzonense*, which were isolated from hot springs in Iceland (Zillig et al., 1983) and Kamchatka (Toshchakov et al., 2015). Representatives of *Thermofilaceae* were thought to be commensals, growing on peptides and simple sugars and requiring undetermined growth factors provided by other crenarchaea (*Thermoproteus*, *Desulforococcus*, *Fervidococcus*, and *Pyrobaculum*, in particular) because the genes of many protein contribute to the biosynthetic pathways for purines, amino acids, and cofactors were absent in their genomes (Anderson et al., 2008). On the other hand, it was shown that the *Thermofilum* species can grow lithotrophically, gaining energy from carbon monoxide (Kochetkova et al., 2019). Moreover, Toshchakov et al. (2015) revealed that *T. uzonense* is capable of growing on two polysaccharides: starch and glucomannan. The growth experiments correlated with *T. uzonense*’s genomic analysis, during which the genes encoding various GHs, including amylases and mannanases, were found. Moreover, in genomes of *T. pendens* (Anderson et al., 2008) and *T. uzonense* (Toshchakov et al., 2015), determinants of cellulose degradation were observed, but no evidence of positive growth on cellulose was shown. *Thermofilum* sp. strain 1505 grew on Avicel (Kochetkova et al., 2019), but its growth was weak, and no additional evidence was obtained to support it. The novel species *T. adornatum* 1910b^T^, which is described here, is able to utilize various carbohydrates, including polysaccharides such as starch, beta-glucan and cellulose (both amorphous and microcrystalline). It is worth mentioning that the final cell yields of the three crenarchaeal species previously reported as capable of growing on cellulose, *Desulforococcus fermentans*, *Thermogladius calderae* and *Thermofilum* sp. strain 1505, were only 1.5–3 times higher than in the control experiment (the same medium without cellulose) (Perevalova et al., 2005; Kochetkova et al., 2016, 2019). This may suggest that the growth on cellulose was determined by the presence of beta-glucosidases, which acted non-specifically, or that the optimal growth conditions were not ascertained. By contrast, the growth of strain 1910b^T^ on cellulose was 10 times higher when compared to a basal medium without cellulose (Figure 1), which cannot be explained by the non-specific action of other enzymes. Moreover, in the case of 1910b^T^, these growth experiments were supported by several activity-based approaches as well as genomic and proteomic analyses.

Native enzyme activities were measured in *T. adornatum* 1910b^T^ cells, which were grown on Avicel using the DNS approach. No enzyme activities were detected in the cell-free culture broth, which implies the enzymes were bound to the cell‘s envelopes via a lipid anchor or a C-terminal transmembrane region or by other mechanisms (Szabo and Pohlschroder, 2012). Cellulases, anchored to the cells and the same time bound to its substrate give additional advantage to cellulolytic microorganism since it will be the first to consume the hyrolysis products (Lu et al., 2006). The active cellulases were washed using various buffers and chaotropic agents, and the highest reduced sugar releasing rates were associated with surface proteins that were solubilized by urea or Triton X 100 (Table 2). In comparison to AMC, a sodium salt of CMC resulted in a higher rate of reducing sugars formation that can be explained by the higher solubility and accessibility of the latter. An absence of cellulase activities in the cells grown on pyruvate indicates that the cellulases of strain 1910b^T^ are cellulose induced.

Despite significant deviation in the spectra of polysaccharides utilized by all studied *Thermofilum* strains (Table 1), the number and variation of genomes in the CAZymes genes are quite similar (Supplementary Figure S3). Thus, the variety of utilized substrates might be determined by yet uncharacterized enzymes, and the overall hydrolytic potential of the genus is not completely recognized. This proposition is consistent with about 30% of protein-coding genes without function prediction in all four *Thermofilum* representatives, according to the Integrated microbial genomes (IMG) portal (Supplementary Figure S4). All *Thermofilum*’s genomes encode the GHs of families GH1, GH3, GH4, GH38, GH57, GH65, and GH109. None of these families include currently known cellulases, moreover taking into account that, except for 1910b^T^ (and possibly strain 1505), none of the strains are capable of growing on cellulose, one would not expect the representatives of these GH families to play a significant role in cellulose hydrolysis in 1910b^T^. Despite the fact that both the *T. adornatum* 1910b^T^ and the *T. uzonense* 1807-2^T^ genomes contain the genes of putative mannanases of the GH113 and GH130 families (*N186_RS00270* and *N186_RS07230* in 1910b^T^ and *MA03_02300* and *MA03_02580* in the 1807-2 genomes, respectively), only *T. uzonense* can grow on glucomannan (Toshchakov et al., 2015); thus, their function in 1910b^T^ is unclear. Still, it is unlikely that these enzymes are involved in cellulose hydrolysis. To our surprise, we were unable to find homologs of a putative cellulase (GH12 family) gene previously found in the *T. pendens* genome (Anderson et al., 2008) in *T. adornatum* 1910b^T^ genome (Supplementary Figure S3). It is possible that this enzyme has a different function because *T. pendens* Hrk5^T^ was not shown to grow on cellulose. Thus, despite the fact that *T. adornatum* 1910b^T^ grows on cellulose, producing cell-bound cellulases, neither genes that encode known cellulases nor GHs distantly related to beta-acting enzymes and exclusively present in *T. adornatum* 1910b^T^ were found in its genome. This led to the proposal that unknown cellulose-degrading enzymes were involved. A more detailed analysis of genomic data as well as an evaluation of genes, which are upregulated during growth on Avicel using proteomics, revealed four candidate endoglucanases (Table 3). Three of them had higher cellulose/control riBAQ ratios than the threshold. Despite the fact that the cellulose/control riBAQ ration for Cel25 protein was lower than the threshold, it had a detectable GH domain and its gene was co-located with another candidate, *Cel30*, which encoded the only extracellular enzyme among the four candidates. In practice, one should be aware that protein localization prediction servers can still be highly inaccurate with archaeal sequences due to the limited amount of experimentally verified data (Bagos et al., 2009). Two of candidate proteins (Cel25 and Cel40) possessed GH1 and GH3 domains, both families contain enzymes with exo-glucanase activities, which may be involved in cellulose degradation (Varghese et al., 1999; Yernool et al., 2000). Two other proteins (Cel45 and Cel30) had no detectable homologs with predicted functions. The proteins Cel40 and Cel45, which were predicted to be intracellular but had high cellulose/control riBAQ ratios, and their genes were co-located. The four candidate cellulase genes were heterologously expressed in *E. coli* BL21 (DE3), and the respective activities of the recombinant proteins were measured. Both DNS activity measurements and TLC of the products of CMC, AMC, and cellobiose hydrolysis suggest that all four recombinant enzymes participated in cellulose degradation. Among them, the Cel25 and Cel30 proteins were likely relatively low-activity exoglucanases with even lower side activities (endoglucanase and beta-glucosidase). Due to its high activity on CMC as well as inability of hydrolizing or producing cellobiose, the Cel45 protein was an endoglucanase. Surprisingly, Cel40 had the highest activity against both CMC and cellobiose, indicating its lesser dependence on the substrate’s length. This observation is in accordance with the most variable set of detectable cellooligosaccharides (Supplementary Figure S2) produced during the action of Cel40. It is probable that both enzymes, Cel40 and Cel45, act synergistically and that their genes are coherently regulated by the presence of a substrate. The synergistic action of the GHs of various substrate specificities and affinities is well known for multienzyme complexes, as cellulosomes (Bayer et al., 2008) or SUS (Shipman et al., 2000), as well as for single polypeptides containing several domains of various functions (e.g., Gavrilov et al., 2016), which degrade huge, rigid or complex polysaccharides. Taking into account that (1) recombinant Cel25 and Cel30 were much less active than Cel40 and Cel45; (2) their gene expressions were (almost) not induced by the presence of CMC, according to proteomic analysis; and (3) the cellulolytic activity of *T. adornatum* 1910b^T^’s SPFs was inducible, we suggest that Cel40 and Cel45 play a major role in cellulose degradation, especially in crucial steps of intramolecular hydrolysis. We suggest that Cel25 and Cel30, on the other hand, are only accessory enzymes. Further work is needed to verify this.

An analysis of these 4 enzymes gene distribution within the *Thermofilum* genomes revealed that *Cel25* and *Cel40* were present in all of them, and they were inherited vertically because the pairwise identities of *Cel25* and *Cel40* were similar to the Average Amino-acid Identity values (Supplementary Figure S5). In their turn, *Cel30* and *Cel45* were present only in the *T. adornatum* strain 1910b^T^ and strain 1505 genomes (Supplementary Table S5). From one standpoint, this observation strongly supports the major role of *Cel45* in cellulose hydrolysis. From another, this observation makes it unclear why strain 1505 only weakly grew on Avicel. The most probable answers have to do with the differences in regulation and/or transport of cellooligosaccharides between these two strains. The incompleteness of the 1505 growth experiments cannot be disregarded.

Thus, *Thermofilum adornatum* 1910b^T^ is the first *Thermofilum* representative capable of cellulose degradation. This was determined by a set of four GHs, two of which represented GH families with previously unknown cellulase activities. The other two were known as hypothetical proteins. The enzymes seem to have different roles in cellulose hydrolysis by strain 1910b^T^ due to differences in substrate specificities as well as various potential mechanisms of secretion, which can affect their localizations and thus their modes of introduction to their substrates.

### Description of *Thermofilum adornatum* sp. nov.

*Thermofilum adornatum* (a.dor.na’tum N.L. neut. adj. *adornatum*, often adorned with clubs at the ends of its cells).

Cells are thin filaments that are 0.15 μm in width and 2–20 μm in length with one flagellum. It is a strict anaerobe. Its temperature, pH and NaCl ranges for growth are 50–95°C, 5.3–8.5 and 0–2.5%, respectively. Its optimal growth conditions include a temperature of 80°C, a pH of 5.5–6.0 and an absence of NaCl. It grows chemoorganoheterotrophically on peptone, yeast extract, AMC, microcrystalline cellulose (Avicel), β-glucan, starch, cellobiose, glucose, lactose, mannose and pyruvate. It does not utilize tryptone, casein, α-cellulose, CMC, lichenan, gelatin, chitin, chitosan, xylan, keratin, mannan, glucomannan, glycerol, sucrose, maltose, xylose, and arabinose. Yeasts extract and CBF of *Desulfurococcus*, *Pyrobaculum* or *Fervidococcus* are required for its growth. The type strain is 1910b^T^ (= DSM 28063^T^ = JCM 19809^T^), which was isolated from a Kamchatkan hot spring (Russia). The genome size is 1.75 Mb. The G + C content of its DNA is 46.5 mol. %. The genome sequence of the strain is deposited in GenBank and IMG under the accession numbers CP006646 and 2554235458, respectively.

## Data Availability Statement

The datasets generated for this study can be found in the Strain 1910bT was deposited in DSMZ (German Collection of Microorganisms and Cell Cultures) with the designation DSM 28063, and it was also deposited in JCM (Japanese Collection of Microorganisms) with the designation JCM 19809. The genome was deposited in Genbank under the accession numbers CP006646 and IMG 2554235458.

## Author Contributions

KZ, TK, and IK conceived the study. KZ and TK performed the microbiological and biochemical experiments. KZ, UP, and IK did bioinformatics analyses. RZ, KZ, and UP performed the proteomics. OP isolated the archaeon into a pure culture. All authors were involved in writing and reviewing the manuscript.

## Conflict of Interest

The authors declare that the research was conducted in the absence of any commercial or financial relationships that could be construed as a potential conflict of interest.

## Footnotes

1 www.cazy.org

2 http://hmmer.org/

3 https://www.maxquant.org/

4 https://bitbucket.org/Ulyana_Piunova/thermofilum-adornatum-1910b/src/master/
