# Supplementary material for: Novel Hyperthermophilic Crenarchaeon Thermofilum adornatum sp. nov. Uses GH1, GH3, and Two Novel Glycosidases for Cellulose Hydrolysis
Source: Front Microbiol. 2020 Jan 10;10:2972. doi: 10.3389/fmicb.2019.02972 (PMC6965361; doi:10.3389/fmicb.2019.02972)
Supplement: Supplementary file 1 [file Data_Sheet_1.pdf]

Supplementary Materials

S1 TABLE | Designed primers for target proteins.

Protein ID	Primer ID	Sequence
Cel25	TA_Nested_225F	5'CCCGGACTAGAAAGGCAGAG
Cel25	TA_TECH_225F	5'GGTGATGATGATGACAAGATGAGTCAACAAATAATTGAAGAATTATTG
Cel25	TA_TECH_225R	5'GGAGATGGGAAGTCATTACTAGTTTCTAAAGGTTTTTTCCTCG
Cel30	TA_TECH_230F	5'GGTGATGATGATGACAAGGACATGGTTGACTCCAAGAAAATAACG
Cel30	TA_TECH_230R	5'GGAGATGGGAAGTCATTAGGGTCCTCTGCCTCCACC
Cel40	TA_TECH_340F	5'GGTGATGATGATGACAAGATGGTTAGAAAGGAATTCCCTGAG
Cel40	TA_Nested_340R	5'GCAACAGGTCGGATTGTTCG
Cel40	TA_TECH_340R	5'GGAGATGGGAAGTCATTATCATATATTTTCACTGCTATGTTTGTTG
Cel45	TA_TECH_345F	5'GGTGATGATGATGACAAGATGACCAAAACCATAGCGGTAG
Cel45	TA_TECH_345R	5'GGAGATGGGAAGTCATTATCACTGTTTGTCAATGTTTGG

S2 TABLE | CAZymes, identified in *T. adornatum* 1910b^T genome.

#Gene	dbCan 2.0 function prediction	Pfam domains organization	Probable function	Signal peptide	Transmembrane helices
N186_RS00225	GH3	GH3-GH3-Fn3	β-glucosidase	-	
N186_RS00270	GH113	ND	β-mannase	+	1
N186_RS00340	GH1	GH1	Putative β-glucosidase	-	
N186_RS00420	GT35	GT1+phosphorylase	Starch phosphorylase	-	
N186_RS00845	CE1	S33	Putative esterase	-	3
N186_RS01315	GT21	GT2	UDP-Glc: ceramide β-glucosyltransferase	-	4
N186_RS01555	GH57	GH57	Alpha-amylase	-	
N186_RS01670	GT2	GT2	Cellulose/polysaccharide-synthase	-	5
N186_RS01830	GT27	GT2	Polypeptide α-N-acetylgalactos-aminyltransferase	-	3
N186_RS01850	GH57	GH57	Alpha-amylase	-	
N186_RS04390	GT66	STT3	Oligosaccharyl transferase	-	14
N186_RS04780	GH109	GFO_IDH_MocA	α-N-acetylgalactosaminidase	-	
N186_RS05430	GT5	GT5	Glycogen synthase	-	
N186_RS05435	GH57	GH57-DuF1925-DuF1926	Alpha-amylase	-	
N186_RS06215	GH109	GFO_IDH_MocA	α-N-acetylgalactosaminidase	-	+
N186_RS06465	CE9	Amidohydro_1	N-acetylglucosamine-6-phosphate deacetylase	-	
N186_RS06480	GH109	GFO_IDH_MocA	α-N-acetylgalactosaminidase	-	+
N186_RS06555	GH1	GH1	Beta-galactosidase	-	
N186_RS06910	CE14	PIG-L	Diacetylchitobiose deacetylase	-	
N186_RS07180	AA4	FAD_binding domain - FAD-oxidase	Vanillyl-alcohol oxidase	-	
N186_RS07230	GH130	GH130	β-1,4-mannosylglucose phosphorylase	-	
N186_RS07265	GH65	GH65	Kojibiose phosphorylase	-	1
N186_RS07400	GH38	GH38-Middledomain-	Alpha-mannosidase	-	

		GH38C			
N186_RS07730	GT2	GT2		-	4
N186_RS07735	GT2	GT2		-	3
N186_RS07745	GT2	GT2		-	3
N186_RS07750	GT2	GT2	Glycosyl transferases involved	-	
N186_RS07755	GT4	GT1	in cell wall biosynthesis	-	4
N186_RS07920	GT2	Glyco_tranf_2_3		-	4
N186_RS07935	GT2	GT2		-	
N186_RS07940	GT4	GT1		-	
N186_RS08040	GH57	GH57-GlucodextranC	Alpha-amylase	-	1
N186_RS08045	GH13_20	Alpha-amylase	Alpha-amylase	-	
N186_RS08485	GT4	GT1	Putative glycosyl transferase	-	
N186_RS08540	GH4	GH4-GH4	Alpha-glucosidase	-	
N186_RS08615	GH16	GH16	Putative endo-beta1-3 glucanase	-	
N186_RS08795	CE7	Hydrolase_4	Cephalosporin-C deacetylase	-	
N186_RS09030	GT2	Glyco_tranf_2_3	Putative glycosyl transferase	-	8
N186_RS09365	CE7	Hydrolase_4	Cephalosporin-C deacetylase	+	8

ND - not detected by the algorithm.

S3 TABLE | The genes with high ratio of cellulose/control riBAQ values.

riBAQ_cell_mean/ riBAQ_contr_mean	Gene ID	Function	Signal peptides (Phobius)	Signal peptides (TatP)	Signal peptides (SignalP)	Transmembrane helices (Phobius)	Transmembrane helices (TMHMM)
∞	*N186_RS00055*	AbrB/MazE/SpoVT_family_DNA-binding_domain-containing_protein_	NO	NO	NO	0	0
∞	*N186_RS00150*	ECF_transporter_S_component	NO	NO	NO	4	4
∞	*N186_RS00325*	ABC_transporter_permease_	YES	NO	NO	5	6
∞	*N186_RS01545*	hypothetical_protein	NO	NO	NO	11	11
∞	*N186_RS01575*	DUF429_domain-containing_protein_	NO	NO	NO	0	0
∞	*N186_RS01745*	ModE_family_transcriptional_regulator	NO	NO	NO	0	0
∞	*N186_RS01770*	hypothetical_protein	NO	NO	NO	4	4
∞	*N186_RS01865*	hypothetical_protein	NO	NO	NO	0	0
∞	*N186_RS02535*	hypothetical_protein	NO	NO	NO	0	0
∞	*N186_RS02740*	30S_ribosomal_protein_S30e	NO	NO	NO	0	0
∞	*N186_RS03045*	flap_endonuclease-1	NO	NO	NO	0	0
∞	*N186_RS03145*	Kae1-associated_kinase_Bud32_	NO	NO	NO	0	0
∞	*N186_RS03210*	hypothetical_protein	NO	NO	NO	0	0
∞	*N186_RS03455*	hypothetical_protein	NO	NO	NO	0	0
∞	*N186_RS03880*	hypothetical_protein	NO	NO	NO	0	0
∞	*N186_RS04130*	hypothetical_protein	NO	NO	NO	0	0
∞	*N186_RS04620*	hypothetical_protein	YES	NO	YES	0	1
∞	*N186_RS04695*	hypothetical_protein	YES	NO	YES	0	1
∞	*N186_RS04830*	hypothetical_protein	NO	NO	NO	0	0
∞	*N186_RS04845*	hypothetical_protein	NO	NO	NO	0	0
∞	*N186_RS05150*	hypothetical_protein	NO	NO	NO	1	0
∞	*N186_RS05800*	DUF91_domain-containing_protein_	NO	NO	NO	0	0
∞	*N186_RS05960*	hypothetical_protein	NO	NO	NO	1	1
∞	*N186_RS06210*	hypothetical_protein	NO	NO	NO	1	1
∞	*N186_RS06620*	hypothetical_protein	NO	NO	NO	3	3
∞	*N186_RS07105*	radical_SAM_protein_	NO	NO	NO	0	0
∞	*N186_RS07225*	NUDIX_domain-containing_protein_	NO	NO	NO	0	0
∞	*N186_RS07435*	hypothetical_protein	NO	NO	NO	0	0
∞	*N186_RS07490*	hypothetical_protein	NO	NO	NO	0	0
∞	*N186_RS07625*	hypothetical_protein	NO	NO	NO	5	3
∞	*N186_RS07905*	hypothetical_protein	NO	NO	NO	0	0
∞	*N186_RS08260*	ABC_transporter_permease_	NO	NO	NO	4	4
∞	*N186_RS08445*	hypothetical_protein	NO	NO	NO	0	0
∞	*N186_RS09020*	hypothetical_protein	NO	NO	NO	0	0
50,90718296	*N186_RS00335*	hypothetical_protein	NO	YES	NO	2	2
44,80889526	*N186_RS00340*	glycoside_hydrolase_family_1_protein	NO	NO	NO	0	0

41,44190978	*N186_RS00330*	_<br>ABC_transporter_permease_	NO	NO	NO	6	6
36,10994726	*N186_RS00065*	hypothetical_protein	NO	NO	NO	1	0
25,16557729	*N186_RS09195*	hypothetical_protein	YES	NO	NO	0	1
18,64521484	*N186_RS00535*	ATP-cone_domain-containing_protein_	NO	NO	NO	0	0
17,73156259	*N186_RS08075*	hypothetical_protein	NO	NO	NO	1	1
14,76535538	*N186_RS07325*	hypothetical_protein	NO	NO	NO	1	1
13,59589106	*N186_RS09360*	hypothetical_protein	YES	NO	YES	0	1
13,19833686	*N186_RS03365*	radical_SAM_protein_	NO	NO	NO	0	0
13,1514073	*N186_RS06530*	hypothetical_protein	NO	NO	NO	8	7
12,02077174	*N186_RS05940*	hypothetical_protein	NO	NO	NO	0	0
11,70440609	*N186_RS01230*	ABC_transporter_ATP-binding_protein	NO	NO	NO	0	0
11,30125896	*N186_RS00340*	carboxypeptidase_regulatory-like_domain-containing_protein	YES	NO	NO	1	2
10,50891896	*N186_RS03790*	hypothetical_protein	NO	YES	NO	0	0
10,28421264	*N186_RS03050*	hypothetical_protein	NO	NO	NO	0	0
9,255949394	*N186_RS04400*	50S_ribosomal_protein_L44e	NO	NO	NO	0	0
8,824295508	*N186_RS04590*	hypothetical_protein	NO	NO	NO	0	0
8,76930959	*N186_RS07960*	hypA_hydrogenase_nickel_incorporation_protein_	NO	NO	NO	0	0
8,740729267	*N186_RS07610*	ATP-binding_protein	NO	NO	NO	0	0
7,925999569	*N186_RS02040*	50S_ribosomal_protein_L32e	NO	NO	NO	0	0
7,7182652	*N186_RS02905*	DUF87_domain-containing_protein_	NO	NO	NO	0	0
7,664465571	*N186_RS03100*	AbrB_family_transcriptional_regulator	NO	YES	NO	0	0
6,928690807	*N186_RS02215*	hypothetical_protein	NO	NO	NO	0	0
6,801554103	*N186_RS02075*	DUF99_domain-containing_protein_	NO	NO	NO	0	0
6,728909752	*N186_RS03885*	PIN_domain-containing_protein_	NO	NO	NO	0	0
6,71403197	*N186_RS06460*	phosphoenolpyruvate--protein_phosphotransferase	NO	NO	NO	0	0
6,673026383	*N186_RS04655*	hypothetical_protein	NO	YES	NO	0	0
6,514179103	*N186_RS04310*	hypothetical_protein	NO	NO	NO	0	0
6,39210034	*N186_RS08505*	hypothetical_protein	YES	YES	NO	1	2
6,273899627	*N186_RS03950*	hypothetical_protein	NO	YES	NO	7	6
5,705866812	*N186_RS06650*	sugar_ABC_transporter_permease	NO	NO	NO	6	6
5,66924546	*N186_RS05325*	DUF126_domain-containing_protein_	NO	NO	NO	0	0
5,581344373	*N186_RS00730*	hypothetical_protein	NO	NO	NO	14	14
5,234149776	*N186_RS00050*	PIN_domain-containing_protein_	NO	NO	NO	0	0
5,070287082	*N186_RS07535*	hypothetical_protein	NO	NO	NO	0	0
5,003085771	*N186_RS00165*	hypothetical_protein	NO	NO	NO	5	5
4,992367763	*N186_RS03980*	hypothetical_protein	NO	YES	NO	0	0
4,956264834	*N186_RS08700*	nucleotidyltransferase_domain-containing_protein_	NO	NO	NO	0	0
4,842324684	*N186_RS06020*	ECF_transporter_S_component	NO	NO	NO	5	5

4,826872668	*N186_RS06380*	hypothetical_protein	NO	NO	NO	14	14
4,813850867	*N186_RS00640*	aldehyde_ferredoxin_oxidoreductase_	NO	NO	NO	0	0
4,632599183	*N186_RS06365*	hypothetical_protein	NO	NO	NO	5	5
4,473133648	*N186_RS08245*	hypothetical_protein	NO	NO	NO	4	4
4,40893768	*N186_RS04580*	hypothetical_protein	NO	NO	NO	0	0
4,359956673	*N186_RS02420*	hypothetical_protein	NO	NO	NO	0	0
4,152293658	*N186_RS05330*	DUF521_domain-containing_protein_	NO	NO	NO	0	0
4,141215819	*N186_RS05460*	sugar_phosphate_isomerase/epimerase_	NO	NO	NO	0	0
4,11527964	*N186_RS06280*	hypothetical_protein	NO	NO	NO	2	2
4,060256849	*N186_RS05405*	hypothetical_protein	NO	NO	NO	0	0
4,050006725	*N186_RS07070*	ABC_transporter	NO	NO	NO	8	6
4,016790096	*N186_RS03095*	hypothetical_protein	NO	NO	NO	1	0
3,99130395	*N186_RS05750*	hypothetical_protein	NO	NO	NO	6	6
3,976466372	*N186_RS04185*	hypothetical_protein	NO	NO	NO	7	5
3,9277433	*N186_RS09025*	hypothetical_protein	YES	YES	NO	0	0
3,901796303	*N186_RS01080*	hypothetical_protein	NO	NO	NO	0	0
3,803695664	*N186_RS09160*	ATP-binding_protein	NO	NO	NO	0	0
3,766476248	*N186_RS00500*	hypothetical_protein	NO	NO	NO	0	0
3,699971272	*N186_RS00940*	hypothetical_protein	NO	NO	NO	0	0
3,656497266	*N186_RS03040*	thioredoxin_	NO	NO	NO	0	0
3,399808116	*N186_RS05775*	ferrous_iron_transport_protein_A_	NO	NO	NO	0	0
3,393979911	*N186_RS00980*	hypothetical_protein	NO	NO	NO	0	0
3,280690398	*N186_RS00395*	ZIP_family_metal_transporter	NO	NO	NO	5	5
3,182963414	*N186_RS00230*	hypothetical_protein	YES	NO	NO	0	1
2,988083312	*N186_RS06755*	hypothetical_protein	NO	NO	NO	0	0
2,980025618	*N186_RS02745*	hypothetical_protein	NO	NO	NO	0	0
2,965130739	*N186_RS09185*	DUF554_domain-containing_protein_	NO	NO	NO	6	5
2,931432391	*N186_RS02230*	50S_ribosomal_protein_L13	NO	NO	NO	0	0
2,930210825	*N186_RS00870*	QueT_transporter_family_protein	NO	NO	NO	8	8
2,928285052	*N186_RS05915*	hypothetical_protein	NO	NO	NO	5	5
2,902997747	*N186_RS00725*	hypothetical_protein	NO	NO	NO	5	5
2,893128599	*N186_RS07315*	hypothetical_protein	NO	NO	NO	6	6
2,833081547	*N186_RS05845*	hypothetical_protein	NO	NO	NO	0	0
2,820225251	*N186_RS06170*	hypothetical_protein	NO	NO	NO	0	0
2,810878891	*N186_RS01510*	hypothetical_protein	NO	NO	NO	0	0
2,801183439	*N186_RS00035*	hypothetical_protein	YES	NO	NO	0	0
2,737589876	*N186_RS01775*	ADP-ribose-binding_protein	NO	NO	NO	0	0
2,722919397	*N186_RS05115*	prenyltransferase_	NO	NO	NO	8	8
2,696439045	*N186_RS08740*	energy-coupling_factor_transporter_transmembrane_protein_EcfT	NO	NO	NO	5	5
2,685166778	*N186_RS06355*	hypothetical_protein	NO	NO	NO	3	3
2,634314761	*N186_RS04380*	hypothetical_protein	NO	NO	NO	0	0

2,569769831	*N186_RS06830*	hypothetical_protein	NO	NO	NO	0	0
2,54776169	*N186_RS07865*	hypothetical_protein	NO	NO	NO	0	0
2,523111874	*N186_RS02835*	hypothetical_protein	NO	NO	NO	0	0
2,522567586	*N186_RS08615*	hypothetical_protein	NO	NO	NO	0	0
2,469024407	*N186_RS05840*	DUF2208_domain-containing_protein_	YES	NO	NO	3	4
2,452516161	*N186_RS08455*	CPBP_family_intramembrane_metalloprotease	NO	NO	NO	5	4
2,437141385	*N186_RS08650*	metal-dependent_hydrolase	NO	NO	NO	0	0
2,422009486	*N186_RS01215*	hypothetical_protein	NO	NO	NO	4	4
2,40147751	*N186_RS08895*	hypothetical_protein	NO	NO	NO	0	0
2,392978405	*N186_RS08020*	energy-coupling_factor_transporter_transmembrane_protein_EcfT	NO	NO	NO	6	5
2,321621631	*N186_RS01020*	hypothetical_protein	NO	NO	NO	4	4
2,308198158	*N186_RS07155*	hypothetical_protein	NO	NO	NO	0	0
2,288135134	*N186_RS03610*	hypothetical_protein	NO	NO	NO	5	5
2,275940129	*N186_RS00905*	hypothetical_protein	NO	NO	NO	0	0
2,271016817	*N186_RS08565*	hypothetical_protein	YES	NO	YES	0	1
2,246847777	*N186_RS07200*	DUF1667_domain-containing_protein_	NO	NO	NO	0	0
2,238154319	*N186_RS05950*	non-canonical_purine_NTP_pyrophosphatase,_RdgB/HAM1_family	NO	NO	NO	0	0
2,204579236	*N186_RS02590*	serine/threonine_protein_phosphatase_	NO	NO	NO	0	0
2,19030528	*N186_RS06855*	hypothetical_protein	NO	NO	NO	0	0
2,174533031	*N186_RS08545*	PIN_domain-containing_protein_	NO	NO	NO	0	0
2,146392372	*N186_RS01500*	hypothetical_protein	NO	NO	NO	0	0
2,11864159	*N186_RS07985*	hypothetical_protein	YES	NO	NO	10	11
2,084776499	*N186_RS07720*	hypothetical_protein	NO	NO	NO	0	0
2,008578376	*N186_RS05475*	D-glycerate_dehydrogenase	NO	NO	NO	0	0

∞ - *when the riBAQ value in control was zero.*

S4 TABLE │ Predicted glycosidases of *T. adornatum* 1910b^{T}.

Gene ID	riBAQ_cel/ riBAQ_contr	Function	Pfam
N186_RS00340 (*Cel40*)	61.7	glycoside hydrolase family 1 protein	GH1
N186_RS06480	6.97	α-N-acetylgalactosaminidase	GFO_IDH_MocA
N186_RS04780	6.7	α-N-acetylgalactosaminidase	GFO_IDH_MocA
N186_RS08540	6.2	alpha-glucosidase	GH4-GH4C
N186_RS08045	4.0	alpha-amylase	GH13
N186_RS01850	3.2	alpha-amylase	GH57
N186_RS05435	1.9	alpha-amylase	GH57-DuF1925-DuF1926
N186_RS00225 (*Cel25*)	1.0	beta-glucosidase	GH3-GH3-X
N186_RS08040	1.0	alpha-amylase	GH57-GlucodextranC
N186_RS06215	0.85	α-N-acetylgalactosaminidase	GFO_IDH_MocA
N186_RS01555	0.8	alpha-amylase	GH57
N186_RS07400	0.8	alpha-mannosidase	GH38-Middledomain-GH38C
N186_RS07230	0.8	β-1,4-mannosylglucose phosphorylase	GH130
N186_RS06555	0.1	beta-galactosidase	GH1
N186_RS00270	0	beta-mannanase	no hits
N186_RS07265	0	kojibiose phosphorylase	GH65
N186_RS08615	0	putative endo-beta1-3 glucanase	GH16

S5 TABLE | Representation of Cel20-45 homologs among four *Thermofilum* genomes.

Query Sequence Name	Gene Id	Gene Name	Genome Id	Genome Name	Query Start Coord	Query End Coord	Subject Start Coord	Subject End Coord	Bit Score	E-value	Identities	Subject Length	
N186_RS00225 N186_RS00225 glycosyl hydrolase 1714559:1716658 forward	2556034304	beta-glucosidase	2554235458	Thermofilum adornatum 1910b	1	699	1	699	1427	0.0	100	699	**Cel25**
N186_RS00225 N186_RS00225 glycosyl hydrolase 1714559:1716658 forward	2628708272	beta-glucosidase	2627853727	Thermofilum adornatum 1505	1	699	1	699	1427	0.0	100	699	
N186_RS00225 N186_RS00225 glycosyl hydrolase 1714559:1716658 forward	639774269	beta-glucosidase (EC:3.2.1.21)	639633064	Thermofilum pendens Hrk 5	13	697	8	699	790	0.0	57	701	
N186_RS00225 N186_RS00225 glycosyl hydrolase 1714559:1716658 forward	2641297020	beta-glucosidase	2639762927	Thermofilum uzonense 1807-2	6	695	5	696	967	0.0	67	702	
N186_RS00230 N186_RS00230 hypothetical protein 1713330:1714448	2556034305	hypothetical protein	2554235458	Thermofilum adornatum 1910b	1	372	1	372	749	0.0	100	372	**Cel30**
N186_RS00230 N186_RS00230 hypothetical protein 1713330:1714448	2628708274	hypothetical protein	2627853727	Thermofilum adornatum 1505	3	372	1	370	744	0.0	100	370	
N186_RS00340 N186_RS00340 glycoside hydrolase family 1 protein	2556034327	beta-galactosidase	2554235458	Thermofilum adornatum 1910b	1	518	1	518	1071	0.0	100	518	**Cel40**
N186_RS00340 N186_RS00340 glycoside hydrolase family 1 protein	2556035604	beta-galactosidase	2554235458	Thermofilum adornatum 1910b	23	506	1	490	427	4,00E-147	47	501	
N186_RS00340 N186_RS00340 glycoside hydrolase family 1 protein	2628708296	beta-galactosidase	2627853727	Thermofilum adornatum 1505	1	518	1	518	1071	0.0	100	518	
N186_RS00340 N186_RS00340 glycoside hydrolase family 1 protein	2628707601	beta-galactosidase	2627853727	Thermofilum adornatum 1505	6	506	2	508	457	2,00E-158	48	519	
N186_RS00340 N186_RS00340 glycoside hydrolase family 1 protein	639774448	glycoside hydrolase, family 1	639633064	Thermofilum pendens Hrk 5	1	510	1	510	713	0.0	65	513	
N186_RS00340 N186_RS00340 glycoside hydrolase family 1 protein	639773887	glycoside hydrolase, family 1	639633064	Thermofilum pendens Hrk 5	6	501	2	503	473	9,00E-165	49	517	
N186_RS00340 N186_RS00340 glycoside hydrolase family 1 protein	2641296933	beta-galactosidase	2639762927	Thermofilum uzonense 1807-2	6	516	2	517	480	1,00E-167	50	517	
N186_RS00345 N186_RS00345 hypothetical protein 1684981:1685214	2556034328	hypothetical protein	2554235458	Thermofilum adornatum 1910b	1	77	1	77	155	5,00E-53	100	77	**Cel45**
N186_RS00345 N186_RS00345 hypothetical protein 1684981:1685214	2628708297	hypothetical protein	2627853727	Thermofilum adornatum 1505	1	77	1	77	155	5,00E-53	100	77	

The analysis was done in the IMG using BLAST/Selected genomes option. BLASTp with 1e-50 cut-off. Cel25, Cel30, Cel40 & Cel 45 amino-acid sequences were used as the queries. The genomes of *T. pendens* strain Hrk 5^T, *T. uzonense* strain 1807-2^T, and *T. adornatum* strains 1505 and $1910b^T$ were used as the subjects.

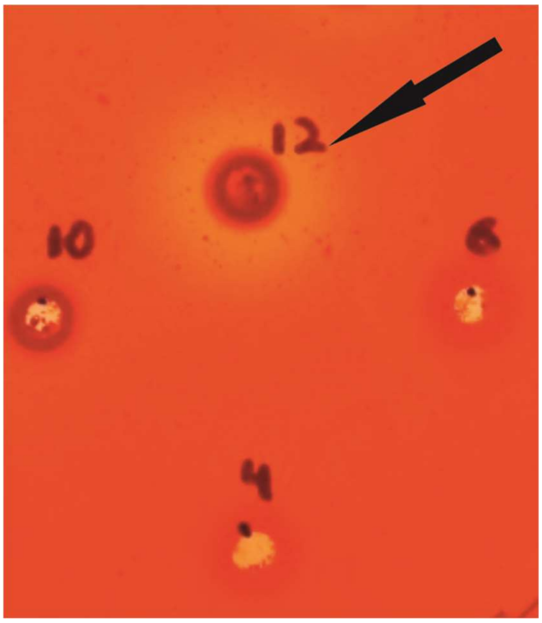

FIGURE S1 | Endoglucanase activity of *T. adornatum* 1910b^T recombinant cellulase Cel45. 10 - crude extract of sonicated *E. coli* BL21 DE3 cells with empty vector (control); 4 - culture broth of *E. coli* cells, containing empty vector (control); 12 – crude extract of sonicated *E. coli* cells, containing recombinant cellulase Cel45; 6 – culture broth of *E. coli* from the same experiment. Substrate, CMC; incubation, 16 h at 80°C. Staining by Congo Red. A halo of CMC hydrolysis is pointed by an arrow.

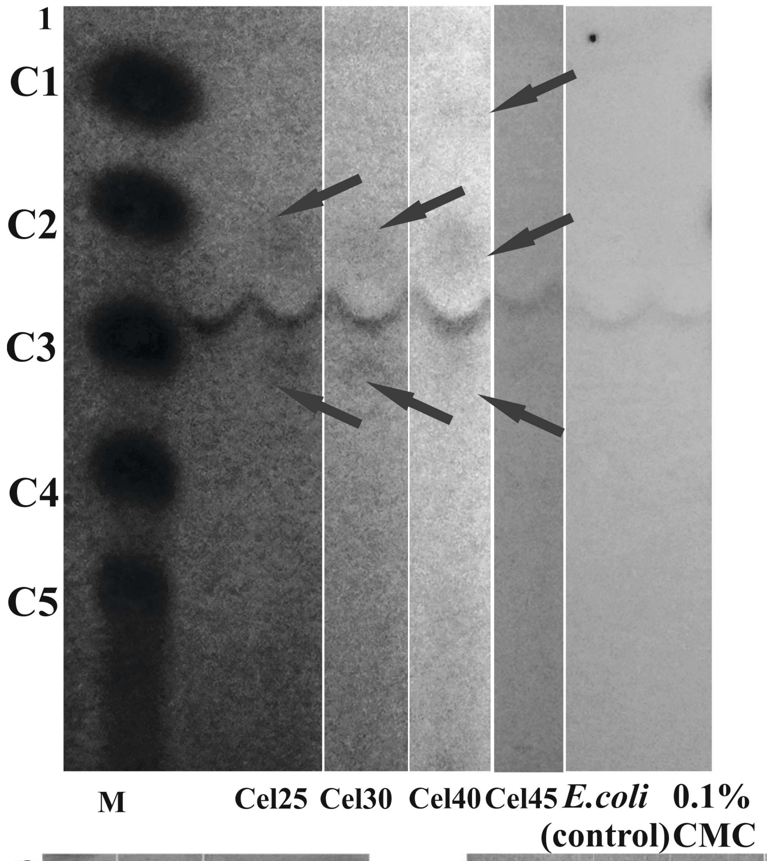
1
C1
C2
C3
C4
C5
M
Cel25
Cel30
Cel40
Cel45
E.coli (control)
0.1% CMC

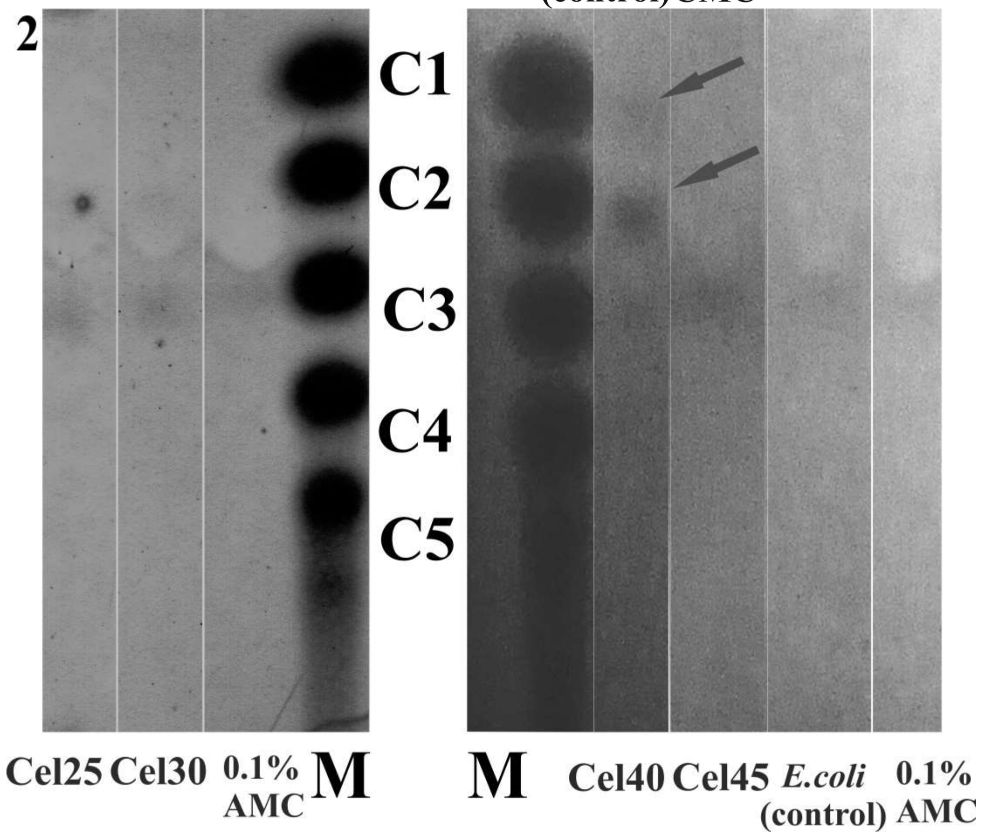
2
C1
C2
C3
C4
C5
Cel25
Cel30
0.1% AMC
M
M
Cel40
Cel45
E.coli (control)
0.1% AMC

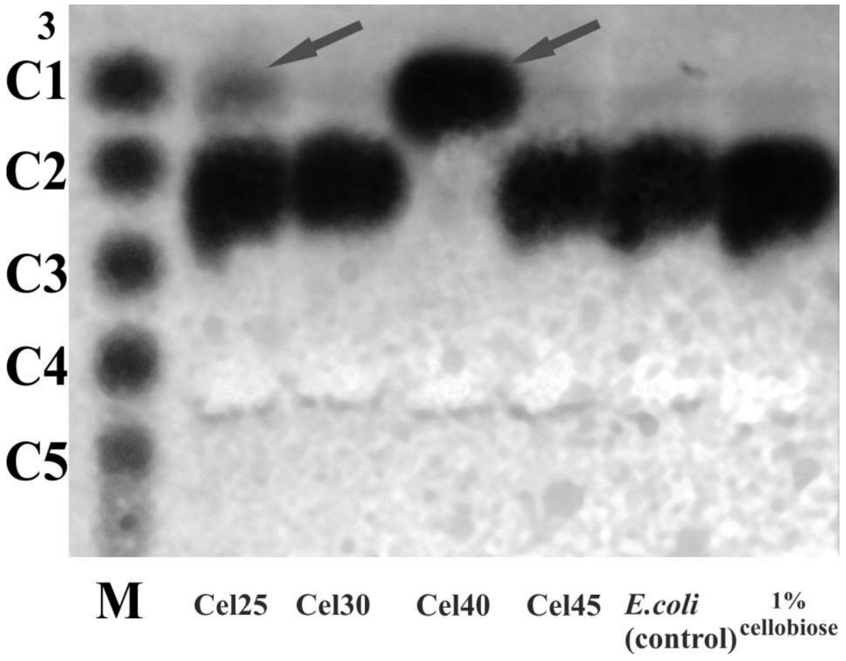

FIGURE S2 │ Degradation of CMC, AMC and cellobiose by the recombinant glycosidases of *T. adornatum* 1910b^T. Formation of glucose and cellooligosaccharides from CMC (1), AMC (2) and cellobiose (3). C - number of glucose units (C1 – glucose; C2 – cellobiose; C3 – cellotriose; C4 – cellotetraose; C5 – cellopentaose); M - a mixture of mentioned above C1-C5 sugars. Arrows indicate the hydrolysis products. Cel25, Cel30, Cel40, Cel45, studied recombinant cellulases of *T. adornatum*.

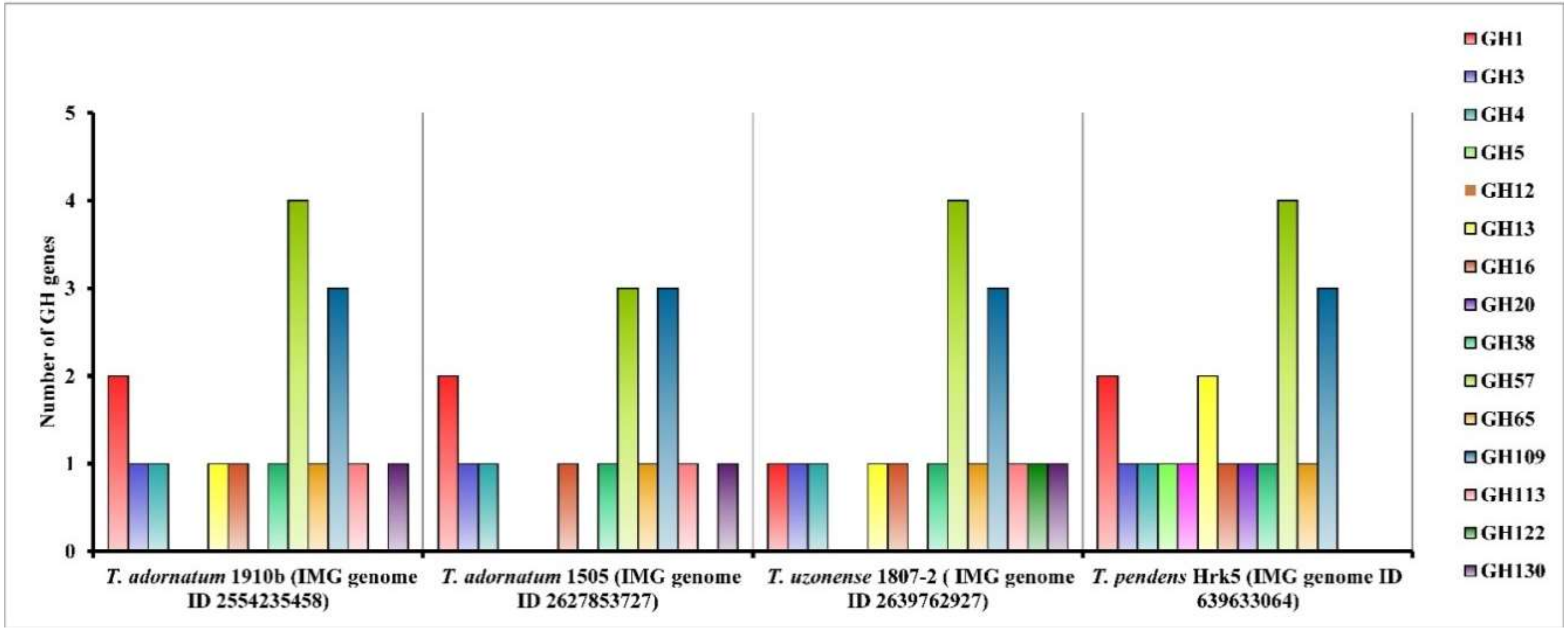

FIGURE S3 | Distribution of glycosidases families' genes among the genomes of cultivated *Thermofilum*.

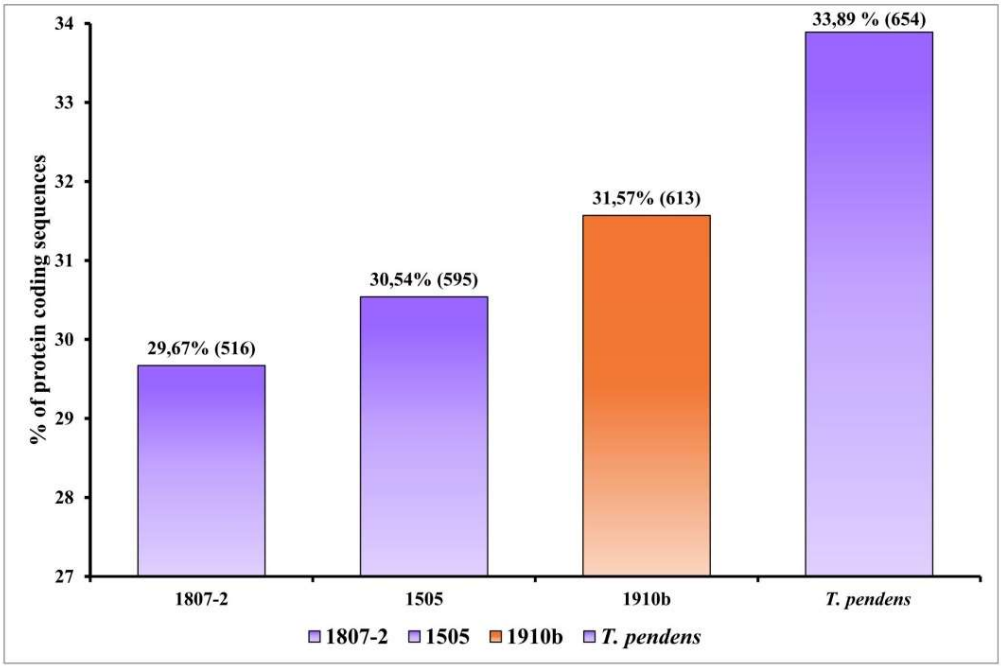

FIGURE S4 | Number of protein-coding genes without function prediction.

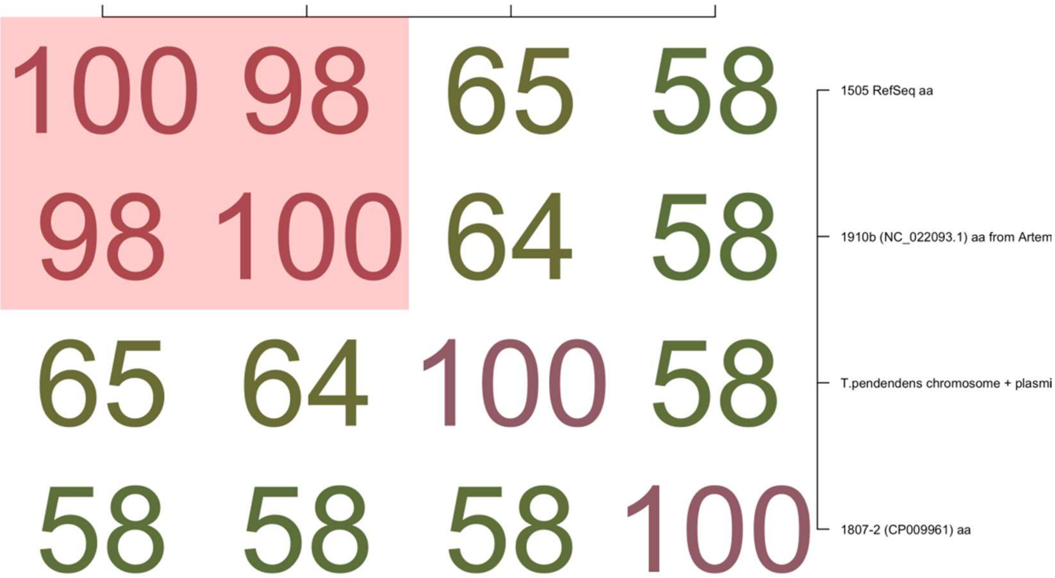

FIGURE S5 | Four *Thermofilum* strains Average Aminoacid Identity Matrix.
